# COMPLICATIONS AFTER HEPATECTOMY

**DOI:** 10.1590/0102-6720202400062e1856

**Published:** 2025-01-20

**Authors:** Claudemiro Quireze, Fabricio Ferreira Coelho, Agnaldo Soares Lima, Hugo Pinto Marques, Martin Palavecino, Timothy Pawlik, Rene Adam, Olivier Soubrane, Paulo Herman, Ricardo Lemos Cotta-Pereira

**Affiliations:** 1 Universidade Federal de Goiás, Department of Gastrointestinal Surgery – Goiânia (GO), Brazil; 2 Universidade de São Paulo, Faculty of Medicine, Department of Gastroenterology – São Paulo (SP), Brazil; 3 Universidade Federal de Minas Gerais, Department of Surgery, Faculty of Medicine – Belo Horizonte (MG), Brazil; 4 Centro Hospitalar Universitário de Lisboa Central, Curry Cabral Hospital, Hepato-Biliary-Pancreatic and Transplantation Centre – Lisbon, Portugal; 5 Hospital Italiano de Buenos Aires, General Surgery Unit – Buenos Aires, Argentina; 6 Ohio State University, Wexner Medical Center, Department of Surgery – Columbus (OH), USA; 7 University Paris-Saclay, AP-HP Paul Brousse Hospital, Cancer and Transplantation Unit, Hepato Biliary Surgery – Villejuif, France; 8 Universite Paris Descartes, Institute Mutualiste Montsouris, Department of Digestive, Oncologic and Metabolic Surgery – Paris, France; 9 Universidade de São Paulo, Faculty of Medicine, Department of Gastroenterology – São Paulo (SP), Brazil; 10 Instituto D’Or de Pesquisa e Ensino, Digestive Surgery Program – Rio de Janeiro (RJ), Brazil.

**Keywords:** Neoplasm metastasis, Hepatectomy, Biliary fistula, Liver failure, Hemorrhage, Colorectal neoplasms, Metástase neoplásica, Hepatectomia, Fistula biliar, Falência hepática, Hemorragia, Neoplasias corretais

## Abstract

Complete removal of metastatic disease and maintenance of an adequate liver remnant remains the only treatment option with curative intent concerning colorectal liver metastases. Surgery impacts on the long-term prognosis and complications adversely affect oncological results. The actual morbidity involving this scenario is debatable and estimated to be ranging from 15% to 50%. Postoperative complications eventually lead to an increase in both mortality rates and tumor recurrence. Biliary fistula and liver failure are the leading complications following liver resection to metastatic colorectal cancer. Prophylactic drainage does not prevent fistulas or hemorrhage. Drainage along with endoscopic intervention and/or surgery may be necessary for grade B and C fistulas. Liver failure is a potentially lethal complication with few therapeutic options. Patient selection and preoperative care are crucial for its prevention.

## RESUMO

A remoção completa da doença metastática e a manutenção de um remanescente hepático adequado continuam sendo o único tratamento com intenção curativa em relação às metástases hepáticas colorretais. A cirurgia impacta no prognóstico de longo prazo e as complicações afetam adversamente os resultados oncológicos. A morbidade real envolvendo esse cenário é discutível e estimada variando de 15 a 50%. Complicações pós-operatórias eventualmente levam a um aumento nas taxas de mortalidade e recorrência do tumor. Fístula biliar e insuficiência hepática são as principais complicações após a ressecção hepática para câncer colorretal metastático. A drenagem profilática não previne fístulas ou hemorragia. A drenagem junto com intervenção endoscópica e/ou cirurgia pode ser necessária para fístulas de graus B e C. A insuficiência hepática é uma complicação potencialmente letal com poucas opções terapêuticas. A seleção do paciente e os cuidados pré-operatórios são cruciais para sua prevenção.

## INTRODUCTION

Complete removal of metastatic disease and maintenance of an adequate liver remnant remains the only treatment option with curative intent concerning colorectal liver metastases.

Surgery impacts on the long-term prognosis and complications adversely affect oncological results^8^. The actual morbidity involving this scenario is debatable and estimated to be ranging from 15 to 50%. Postoperative (PO) complications eventually lead to an increase in both mortality rates and tumor recurrence^2^.

The wide variation on this incidence can be explained by the multiplicity of patient and procedural features. Consequently, an increased morbidity may be expected among patients who are elder, are frail, and had previous preoperative procedures (e.g., biliary interventions). Also, a difficult tumor location has been proved an issue that may increase surgical difficulty and compromise surgical outcomes^1^.

This overall PO morbidity may be split in terms of the most troubling complications confronted. Biliary fistula and posthepatectomy liver failure (PHLF) are the leading causes of complications demanding reoperation in colorectal liver metastasis patients. To improve quality standards in colorectal metastasis liver surgery, Oliver et al. advised that its quality limits should not exceed 10% (biliary fistulas) and 8% (liver failure)^15^.

The aim of this article is to provide a brief review of the leading causes of PO morbidity in the setting of colorectal liver metastases.

## METHODS

Our comprehensive literature search aimed to identify 20 relevant articles in the Medline/PubMed, LILACS, and SciELO databases, mainly from the last decade. Full texts, reviews, systematic reviews, and meta-analyses regarding PO complications in the setting of liver resection and colorectal liver metastases were included.

### Postoperative biliary fistula

Biliary leakage is a major cause of PO morbidity, often demanding diagnostic tests, interventions, and prolonged hospital stay^12,21^.

The International Study Group of Liver Surgery proposed an international consensual definition and grading, after an extensive retrospective research.

Postoperative biliary fistula (POBF) can be stated either as a discharge of intra-abdominal fluid on or after PO day 3 or as the need for relaparotomy or any intervention toward evacuating biliary collections. Increased bilirubin level within these fluids is defined as a concentration at least three times the serum bilirubin (SB) level measured at the same time. POBF can be classified according to severity, as follows:

- POBF grade A: Bile leakage requiring no or little change in patients' management.
- POBF grade B: Bile leakage lasting longer than 1 week or requiring a change in patients' management (e.g., additional diagnostic or interventional procedures) but not reoperation.
- POBF grade C: Bile leakage requiring reoperation.

Many other definitions of POBF are available. Controversy about the cutoff values of bilirubin levels, volume of the fluid, and time intervals after surgery are the basis for discordant opinions.

Spetzler et al. reported a POBF incidence of 2.8, 8, and 3.2% for grades A, B, and C, respectively. Patients from this cohort with colorectal liver metastases had an overall POBF rate of 17.3%. Other series reported incidences ranging from 2.2 to 9.5% (grade A), 3.4 to 27.4% (grade B), and 0 to 4.1% (grade C), revealing that besides gravity, treatment strategies, reoperation policies, and preoperative care may impact its true incidence. Also, previous chemotherapy, preoperative biliary intervention, major hepatectomy, and biliodigestive anastomosis were identified as risk factors that support an increased risk of POBF^19^.

Routine placement of abdominal drains is a practice intended to minimize the consequences of a POBF. Moreover, some advocate that drains would prevent PO intra-abdominal collections, control bile leakages, divert ascites from the wound, and provide early diagnosis of hemorrhage. This widely applied strategy seems more experience-based rather than evidence-based^7^.

Bekki et al. advocated that clinical predictors may be useful for the selective use of abdominal drains. In this study, prophylactic drainage aimed to identify whether biliary fistula or hemorrhage was successful for patients with intraoperative bile leakage, prolonged operation time (³360 min), or blood loss exceeding 650 ml^4^.

A multicentric study by Brooke-Smith et al. indicated that the risk of PO intervention did not change with the routine placement of abdominal drains. In this series, the need for radiological interventions to treat a biloma was observed in 9.2% of intraoperative drainage patient group vs. 5.8% of the no-drain patients. In this cohort, intraoperative drain placement and intraoperative blood loss were independent factors associated with POBF^5^.

There is considerable heterogeneity regarding the studies focusing this subject. Diverse POBF definitions, types of liver resection, etiology of primary tumor, and concomitant biliary reconstruction are frequently addressed along, weakening subsequent systematic reviews and meta-analyses^13^.

Squires et al. focused on the value of preemptive abdominal drainage following a major hepatectomy. In this series, routine drainage was associated with increased POBF risk and 30-day hospital readmission rates^20^.

Recently, Gavriilidis et al. linked the incidence of ascitic leak to the use of abdominal drains, corroborating others that their routine use cannot be warranted^10^.

### Posthepatectomy liver failure

The definition of PHLF, also known as hepatic insufficiency, has also been a controversial question. Its incidence ranges from 1 to 34%. Increased SB level and prolonged prothrombin time (PT) have served as prognostic markers since the former is less likely to be biased following liver resection and the latter is a reliable liver function indicator.

Balzan et al. indicated that, following a hepatectomy, these rates inclined toward normal values on PO day 5. Instead, persistence of PT<50% (international normalized ratio [INR]=1.7) and SB<50 μmol/l (3 mg/dl) on PO day 5 granted a 59% risk of early PO mortality. This concept, known as the "50–50 criteria" on PO day 5, was considered a consistent predictor of PHLF^3^.

Mullen et al. reviewed data from 1059 major (³3 segments) hepatectomies from patients with a normal baseline bilirubin level at three hepato-pancreatico-biliary centers. Focusing on the PO bilirubin rates, a multivariate analysis evidenced that a peak bilirubin level above 7.0 mg/dl was an independent reliable predictor of 90-day liver-related mortality^14^.

Therefore, PHLF is a postoperatively acquired loss in the ability of the liver to maintain synthetic, excretory, and detoxifying functions in the absence of other causes for this clinical and biochemical impairment. This damage is characterized by increased INR values and simultaneous hyperbilirubinemia on or after PO day 5.

These authors also proposed a simplified grading system:

- PHLF grade A: Abnormal laboratory findings requiring no change in patient's clinical management.
- PHLF grade B: Deviation from regular clinical management but without the need for invasive treatment.
- PHLF grade C: Deviation from regular clinical management and requiring invasive treatment.

The etiology of PHLF is usually related to the patient (diabetes, obesity, cholangitis, and malnutrition), surgery (blood loss, inadequate future liver remnant [FLR], major hepatectomy, and transfusion), and prior liver function (steatosis, sinusoidal injury, chemotherapy-associated injury, and hyperbilirubinemia).

Chemotherapy-associated liver injury is a unique risk factor of liver failure. Severe sinusoidal dilation due to oxaliplatin-based treatments is implicated in increased PO morbidity. Interestingly, the association of bevacizumab with these treatments seems to minimize these effects. Steatohepatitis, mainly linked to irinotecan, has also been related to increased PO complications^22^.

Accurate patient selection and planning are crucial to mitigate PHLF. Predictive scoring systems, such as Child-Pugh and Model for End-Stage Liver Disease (MELD), are useful in the preoperative setting but not specifically validated to predict PO failure. Indocyanine green clearance provides a reliable functional estimation, but volumetric assessments are more useful for anticipating liver insufficiency.

Using liver volumetry, estimated future remnants (FLR)³20% are considered safe for liver resections in patients with healthy livers. Instead, an FLR³30% is required for those who had prior extensive chemotherapy and an FLR³40% for cirrhotic ones.

### Therapeutic challenges

A detailed appraisal of treatment is beyond the scope of this paper. Grade C complications are better managed in specialized centers by multidisciplinary teams^9,11^.

Prophylactic drainage does not preclude a POBF or hemorrhage. When in place, there is no consensus about the time for its removal. Percutaneous management of PO bile leaks is considered a safe approach. When drainage alone fails, the patient is at risk of sepsis and death. Endoscopic treatment with sphincterotomy and stenting may be useful for patients with persistent or high-output fistulas and an intact common bile duct. Leakage from isolated segmental bile duct injuries may require a surgical approach^6^.

PHLF implies in the risk of mortality and has limited supportive treatment options. Prevention must be the standard practice. Attention must be paid to patients who are obese, are diabetic, and receive more than six cycles of systemic chemotherapy. Portal vein embolization may be necessary for patients with small FLRs^18^.

There are limited surgical options available for avoiding PHLF. Intraoperative modulation is useful when the portal venous pressure exceeds 20 mmHg. Ischemic preconditioning is another alternative and prevents liver cell death from ischemia-reperfusion injury^16,17^.

## CONCLUSIONS

Biliary fistula and liver failure are the leading complications following liver resection to metastatic colorectal cancer. A prophylactic drainage does not prevent fistulas or hemorrhage. Drainage along with endoscopic intervention and/or surgery may be necessary for grade B and C fistulas. Liver failure is a potentially lethal complication with few therapeutic options. Patient selection and preoperative care are crucial for its prevention.

## AUTHORS' COMMENTS

Hugo Pinto Marques: "About complications, I think there are types of complications that can be related to technical issues,but there are also types of complications, that are essentially related to bad decisions."

Rene Adam: "I would say the volume and functionality of the liver remnant, which is the main factor involved in the possibility of liver insufficiency. And also, the assessment of the disease of the liver by the action of chemotherapy… For me, it's a good evaluation observe the volume of liver remnant, and in doubtful cases, not to hesitate to do PVE of the contralateral side… The best way to prevent this is to be a little bit up the normal volume or the normal ratio between the remnant liverand the body weight of the patient. I would say, over 0.5, much better 0.6 or 0.7 for normal liver. For cirrhotic, we don't know what is ideal ratio, some have advanced to 0.8, but nobody knows. In my view, the best way to prevent is to be a little bit over the limits of 30% of liver remnant and 0.5 ratio of liver remnant and the body weight."

Rene Adam: [effects of chemo on the liver] "Biopsy would be the best one but we don't do it routinely, I think that the level of gamma GT could be a good index. Also, the transaminase when they are slightly elevated could be also an index…. Sometimes, when the patient has received prolonged administration of oxaliplatin we mayhave some lower platelets and some sign of portal hypertension."

Hugo Pinto Marques: "I think we know that six cycles or less has no significant impact…We have some ways to evaluate the impact of oxaliplatin and sinusoidal obstruction syndrome (SOS)… We published that 10 years ago with APRI score, the transaminase is divided by platelet count, and 0.64 is the threshold that you have a chance tohave SOS… Sometimes you have very severe forms with portal hypertension, so you can detect with a big spleen… that's a bad sign."

Martin Palavecino: [Refractory ascites] "We do not see that much in the CRLM, we see in CHC in cirrhotic liver and portal hypertension. Is also an issue when you have open surgery because… you have a trouble with the leaking ascites in the place of the incision. So that's very easier in laparoscopic surgery, because of the small incisions… In patients with small for size syndrome we usually use spironolactone (Aldactone) as the drug to try to decrease the production of ascites."

Olivier Soubrane: [Portal vein thrombosis] "I think there are two situations most often, that are probably technical issues… Divide the portal vein very close to the bifurcation in a transversal way, or complex extended resection, when you have the portal vein kinking… I sent a patient to the CT scan and then go to the operating room… You have to remove the thrombus and probably to do something on the portal vein, including redo anastomosis."

Rene Adam: "It's a technical problem and in my view, this complication occur much more on the left side, when you have done the right extended hepatectomy tosegment one, you have to resected part of the boundary of the portal vein and there is a sort of kinking between the direction of the left portal vein and the portal trunk and sometimes you are at 90 degrees…It is very necessary to have a very good direction of the left remnant liver in the prolongation of the portal trunk… Sometimes you should have to reconstruct with a flap, to do a plasty, or sometimes with a peritoneal patch in a way to make that the remnant portal vein is totally open."

Paulo Herman: [Prevent bile leackage] "We use MRI as a routine, and during the planning we study if there are any anatomical variations and in the end of a major hepatectomy we usually do a cholangiography and a test using injection of saline and air."

Martin Palavecino: "Probably when you have a such a high leak on the first postoperative days is a technical issue… One assuming that this fistula is well drainedby the drainage, we proceed with ERCP. In those situation, sometimes you can deal with it with a stenting or ERCP… Usually we wait four or five days to perform ERCP in order to understand what's happening."

Rene Adam: [Drains] "There are a recent randomized study by a Japanese group showing that even for major hepatectomies there is no evidence for putting a drain routinely…I think I have changed my attitude because I have been teaching that every hepatectomy should be drained, and now I have moved to the other extreme no drain for the majority. I put a drain when it is an extensive hepatectomy of I have some doubt about the possibility of bleeding or biliary fistula. So it is a minority of cases. Suction drain is OK."

Hugo Pinto Marques: "We used to drain a lot, now we tend to drain less and less, but we do drain major hepatectomies, multiple parenchymal sparing hepatectomies… and also when you have a bilioenteric anastomosis routinely."
